# Mapping of catecholaminergic denervation, neurodegeneration, and inflammation in 6-OHDA-treated Parkinson’s disease mice

**DOI:** 10.21203/rs.3.rs-5206046/v1

**Published:** 2024-10-16

**Authors:** Matteo Santoro, Rachel K. Lam, Sarah E. Blumenfeld, Weiqi Tan, Peter Ciari, Emily K. Chu, Nay L. Saw, Daniel Ryskamp Rijsketic, Jennifer S. Lin, Boris D. Heifets, Mehrdad Shamloo

**Affiliations:** Stanford University School of Medicine

**Keywords:** Parkinson’s disease, 6-OHDA, Dopamine, Striatum, Substantia Nigra, Locus Coeruleus, Dopaminergic projections, Stereology cell counting, iDISCO+, LSFM, Cytokines/chemokine, Dopaminergic lesion

## Abstract

Efforts to develop disease-modifying treatments for Parkinson’s disease (PD) have been hindered by the lack of animal models replicating all hallmarks of PD and the insufficient attention to extra-nigrostriatal regions pathologically critical for the prodromal appearance of non-motor symptoms. Among PD models, 6-hydroxydopamine (6-OHDA) infusion in mice has gained prominence since 2012, primarily focusing on the nigrostriatal region. This study characterized widespread tyrosine hydroxylase-positive neuron and fiber loss across the brain following a unilateral 6-OHDA (20 μg) infusion into the dorsal striatum. Our analysis integrates immunolabeling, brain clearing (iDISCO+), light sheet microscopy, and computational methods, including fMRI and machine learning tools. We also examined sex differences, disease progression, neuroinflammatory responses, and pro-apoptotic signaling in nigrostriatal regions of C57BL/6 mice exposed to varying 6-OHDA dosages (5, 10, or 20 μg). This comprehensive, spatiotemporal analysis of 6-OHDA-induced pathology may guide the future design of experimental PD studies and neurotherapeutic development.

## 1 Introduction

Parkinson’s disease (PD) presents pathological characteristics that are challenging to fully replicate in rodent and non-human primate models. PD is a multifaceted disorder encompassing motor and non-motor manifestations.^1^ Neurodegeneration and aggregation of Lewy bodies (LB) extend well beyond the nigrostriatal tract,^2^ affecting various neurotransmitters such as dopamine,^3^ norepinephrine,^4^ serotonin,^5^ and GABA.^6^ Despite the limitations of modeling PD in small animals, the understanding of PD pathophysiology and cellular disease mechanisms have primarily been driven by animal models mirroring certain pathological aspects of the disease. However, such advancements have not translated into definitive treatments to cure or delay neurodegeneration in PD, with current therapeutic approaches primarily focused on symptomatic management.^7^ Though numerous mouse models of PD have emerged over the years to elucidate PD’s cellular, genetic, and environmental aspects,^8–11^ only a handful can effectively serve as platforms for drug screening.^10,12,13^ Notably, most preclinical drug testing relies on PD models that have been successfully used for more than four decades, i.e., systemic injection or local infusion of neurotoxins such as 1-methyl-4-phenyl-1,2,3,6-tetrahydropyridine (MPTP) or 6-hydroxydopamine (6-OHDA) into rodents.^11,14,15^ The neurotoxin 6-OHDA was first discovered in 1959 and employed by Ungerstedt in 1968 in rats as a catecholaminergic denervating agent.^16^ In the present study, we chose the dorsal striatum as the site of infusion to achieve gradual and progressive loss of dopaminergic cells in a retrograde fashion as demonstrated elsewhere^17^ and avoid undesired effects such as high mortality, nonspecific toxicity, and acute neuronal loss arising from targeting regions such as the medial forebrain bundle, the ventricles, and substantia nigra pars compacta (SNpc). Existing reports guide the behavioral and histopathological phenotyping of 6-OHDA lesioned mice.^11,18,19^ For example, Stott et al. noted a swift loss of striatal tyrosine hydroxylase-positive (TH+) fibers, accompanied by TH + cell loss starting on day 6 and microglial proliferation on day 9 after the striatal convection-enhanced delivery (CED) of 10 μg of 6-OHDA.^18^ Similarly, Masini et al. offered comprehensive guidance on pre-and post-operative care to minimize mortality after bilateral infusion of 4 μg of 6-OHDA. They also reported sex differences and cell loss in the SNpc and ventral tegmental area (VTA) regions.^11^

**Figure 1:**
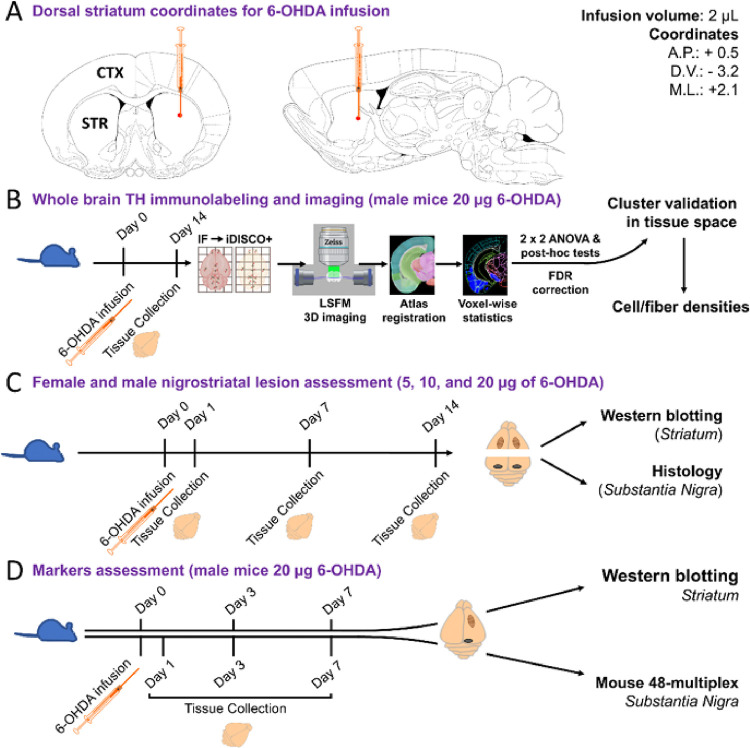
Brain infusion coordinates of 6-hydroxydopamine (6-OHDA) and experimental flowcharts. **A)** 6-OHDA infusion site based on coordinates relative to Bregma in C57BL/6 mice (12–14 weeks old). 2 μl of 6-OHDA or vehicle solution was infused at 0.5 μl per minute. Brain images were adapted from the Paxinos, George, and Keith B.J. Franklin Mouse Brain Atlas (2001). **B**) iDISCO/LSFM flowchart. 6-OHDA (20 μg; n = 10) and vehicle (n = 8) brains were collected 14 days post-infusion. Brain tissue was immunolabeled, cleared via iDISCO+, and imaged with LSFM. 3D autofluorescence images were registered to the Allen brain atlas (ABA), and 3D images of TH immunoreactivity were background subtracted and warped to atlas space. Voxel-wise 2×2 ANOVAs mapped main effects and interactions, and voxel-wise *post hoc* t-tests delineated directional changes in TH labeling. After False Discovery Rate (FDR) correction, clusters of significant voxels were warped to tissue space to validate differences in TH+ fiber and cell densities. **C**) Experimental design for assessing the nigrostriatal lesion extent in male and female cohorts following infusion of 5, 10, and 20 μg of 6-OHDA by convection-enhanced delivery. **D**) Characterizing inflammatory markers involved infusing 20 μg of 6-OHDA in male mice with time points at 3 days post-surgery for western blotting analysis and 1-, 3-, and 7-days post-surgery for measuring cytokines and chemokines via the Luminex 48-plex assay. Additionally, kinase phosphorylation status was determined by Western blot 7 days post-surgery.

The primary aim of our study was to extensively characterize the histopathological and biochemical changes resulting from the unilateral infusion of 6-OHDA into the dorsal striatum (Fig. 1).^20^ In a first for this model, we mapped TH depletion in 3D across the whole brain by imaging immunolabeled solvent-cleared brains with light sheet fluorescence microscopy (LSFM) and extending the quantitative functionality of UNRAVEL^21^ to use voxel-wise maps of differential immunolabeling for precise TH + neuron projection mapping in the regions most severely affected by 6-OHDA. This innovation enabled us to visualize and quantify changes in TH + cell and fiber density unbiasedly in both ipsilateral and contralateral hemispheres of 6-OHDA-treated mice and a vehicle-infused control group. We also extensively assessed differences in neuronal damage between male and female C57BL/6 mice across three different dosages of 6-OHDA. Finally, we evaluated chemokine and cytokine expression in the SNpc while screening for pathologically relevant kinases and inflammatory markers in the striatum. 3D mapping highlighted the widespread loss of TH + projections in both hemispheres two weeks after a high dose of 6-OHDA (20 μg), coupled with compensatory increases in TH expression in projections targeting subcortical regions. Voxel-wise and region-wise analyses indicated that the loss of catecholaminergic neurons is restricted to the SNpc. Our biochemical and 2D histological findings revealed significant disparities between male and female mice, with females exhibiting resistance to the effects of 6-OHDA. In male mice, the lowest dose of 6-OHDA (5 μg) led to gradual striatal fiber loss (with measurements at days 1, 7, and 14), whereas the loss of TH + cells in the SNpc was only gradual for the highest dose (20 μg). Correspondingly, several cytokines and chemokines were upregulated in the SNpc by the highest 6-OHDA dosage, consistent with SNpc degeneration. We also examined the infusion site for inflammatory marker expression and phosphorylation of pro-apoptotic kinases. Glial reactivity was observed early on, and pro-apoptotic signaling appeared specific to DAPK1, suggesting the involvement of this protein kinase in the pathophysiology associated with the loss of SNpc neurons. Our comprehensive characterization of the 6-OHDA mouse model expands our understanding of its pathology and may inform the development and screening of experimental therapeutics for neuroprotective and immunomodulatory treatments for Parkinson’s disease.

## 2 Materials and methods

### 2.1 Animals

Eight-week-old male and female C57BL/6 mice were purchased from The Jackson Laboratory (Bar Harbor, ME). Unless otherwise specified, animals were group-housed (four/five per cage) in environmentally controlled rooms (19.5–21.5°C, 60–65% relative humidity) with a 12-hour reverse light/dark cycle (lights on at 8 p.m.). Mice were held in 100% PET disposable individually ventilated cages (IVC) with a filter cage IVC lid. The cages were docked into IVC Innoracks designed to offer continuous airflow within the cages and dual HEPA-filtered ventilation (Innovive). Cages were filled with ALPHA-dri bedding, and mice had free access to water and food (chow Teklad global 18% protein rodent diet). The Stanford University Administrative Panel approved all procedures and animal husbandry practices for Laboratory and Animal Care (APLAC) and adhered to the US National Institutes of Health Guide for the Care and Use of Laboratory Animals. Our laboratory is also compliant with the Association for Assessment and Accreditation of Laboratory Animal Care (AAALAC) standards and accredited by the AAALAC international accreditation program for the use of animals in research. Annual inspections are conducted throughout our facilities by both APLAC and AAALAC. Paper nestlets and cardboard tubes provided environment enrichment (1 cage change per week). Experimenters involved in histopathology assessment, Western blotting, and immune marker assays were blind to the treatment groups. Mice were identified with numerical IDs. Experimenters and animal caregivers were unblinded during animal surgeries and post-operative care procedures. Data analysis procedures were carried out in an unblinded fashion. A total of 215 mice were used.

### 2.2 6-OHDA procedures

#### 2.2.1 Pre- and post-operative care

Before surgery, mice were acclimatized in the housing facilities for at least a week. Mice were randomized into treatment groups based on their body weight and sex. Differences in the average body weight between groups were less than 1.5 grams. Group housing of 4/5 mice per cage was done to ensure even distribution of the control vehicle and 6-OHDA-infused mice in the same cage. To reduce stress levels, mice were handled for 4 days before surgical procedures. Mice were anesthetized using 4% isoflurane (Isothesia, Henry Schein Animal Health, Dublin, OH) in a mixture of oxygen and compressed air. The toe pinch reflex confirmed full anesthetic induction. Immediately after induction, mice received a subcutaneous (s.c.) injection of USP solution (10 mL/kg) with 5% dextrose (Baxter, Deerfield, IL) and 1 mg/kg Buprenorphine SR^™^ LAB (ZooPharm). Mice were shaved on top of the skull and positioned on a digital stereotaxic frame (David Kopf Instruments), with isoflurane maintained at 1.5–2% throughout the surgery. Eye drops were applied to keep the cornea moisturized, and the skull surface was disinfected 3X with Betadine and 70% ethanol solution. An 8 mm median incision above the skull was topically anesthetized twice with 5 mg/mL lidocaine (Xylocain MPF, Fresenius Kabi, USA). Respiration rate, skin color, and toe-pinch reflex were monitored every 15 minutes, while normothermia was maintained through a heating pad (WPI, ATC2000) connected to a feedback loop rectal probe (WPI). After intracranial convection-enhanced delivery (CED) of 6-OHDA or vehicle, mice were placed in recovery cages and kept warm with heating pads, with free access to 98% water hydrogel (Clear H_2_O) for approximately 5 hours before returning to their home cage. Saline was administered s.c. every day for 5 consecutive days, with wetted food provided throughout the treatment period. Buprenorphine SR^™^ LAB was administered every 48 hours to manage pain. Attrition rates ranged between 5 and 30% depending on the sex and dose of 6-OHDA infused. Pre- and post-surgical care was conducted per the guidelines outlined by Masini et al.^11^

#### 2.2.2 Unilateral convection-enhanced delivery (CED) of 6-OHDA

6-OHDA hydrochloride was from Sigma Aldrich (Cat: H4381 Lot number: MKCG8789). 6-OHDA handling and infusions were carried out following the guidelines and safety procedures described by Stott and Barker.^18^ Using CED, 6-OHDA was infused at one of three dosages: 5 μg (low dose)^22^, 10 μg (medium dose)^18^, or 20 μg (high dose).^17^ Vehicle control mice were infused with USP saline 0.9% containing 0.03% ascorbic acid. Mice were unilaterally infused at a rate of 0.5 μL/min into the dorsal striatum (AP = + 0.5 mm, ML = + 2.1 mm, DV = −3.20 mm from bregma (see Fig. 1A)). The equipment used for CED consisted of a 10 μL airtight syringe (Hamilton), a 33-gauge Hamilton needle, and a microinjection pump (UMP3, WPI) connected to an intelligent touch controller (WPI). After the infusion, the needle was left in place for 5 minutes before being slowly withdrawn. The incision site was closed with tissue adhesive (3M Vetbond Tissue Adhesive, Cat: 1469SB). Before every infusion, the needle and syringe were inspected to ensure accurate delivery of a total volume of 2 μL.

### 2.3 Assessment of 6-OHDA-induced lesion extent

#### 2.3.1 Tissue harvesting

For lesion quantification, animals were sacrificed at selected time points: day 1, day 3, day 7, and day 14 (see Fig. 1). Animals were anesthetized with isoflurane 4% and intracardially perfused with phosphate-buffered saline (PBS). Brain regions designated for western blotting (WB) and multiplex-cytokine/chemokines, including the striatum and substantia nigra, were quickly sub-dissected and snap-frozen. Hindbrains destined for histological assessment of TH + cells in the SNpc were post-fixed for 48 hours in a 4% paraformaldehyde and then cryopreserved in 0.1 M phosphate buffer (PB) containing 30% sucrose.

#### 2.3.2 Striatal semi-quantitative analysis of TH protein levels via WB

Tissue processing, sample preparation, and loading into the gels were conducted as described.^23^ The antibodies used are listed in Table S1. The blots were imaged using the Sapphire Molecular Imager and analyzed using Azure Spot (Azure Biosystems) software.

#### 2.3.3 Measurement of immunoreactive TH + cells in the substantia nigra (mean fluorescence intensity)

Coronal brain sections, 30 μm thick, were stained as free-floating slices. Briefly, sections were washed in 0.1 M PB containing 0.1% Triton-X 100. The sections were then blocked to prevent nonspecific binding using a mixture of 4% normal goat serum (NGS) and 1% bovine serum albumin (BSA) in 0.1 M PB with 0.4% Triton X-100. Slices were incubated with chicken polyclonal anti-TH (1:2000, Abcam, ab76442), washed, and then incubated with donkey anti-chicken polyclonal antibody conjugated with Alexa Fluor 594 (1:250, Jackson Immuno Research, 703-545-155). Sections were washed with 0.1 M PB containing DAPI (2 μg/mL; stock solution: 10 mg/mL, Millipore Sigma), then mounted and coverslipped using polyvinyl alcohol mounting media (Millipore-Sigma, 10981). A blinded operator performed image acquisition at two levels within the SNpc, spaced 740 μm apart, for the ipsilateral and contralateral hemispheres. The sections were visualized with an upright epifluorescence microscope (AxioImager M2, Zeiss), and images were acquired with Stereoinvestigator software, version 10 (MBF Bioscience). Images were quantified using FIJI (Image J version 1.53). Mean grey values were measured in the SNpc and manually segmented based on anatomical references.^24^

#### 2.3.4 Stereological cell counting of nigral TH + neurons

An average of ~ fifty coronal brain sections (30 μm thick) were obtained from the ventral midbrain, covering a region approximately 1.44 mm in length, ranging from −3.88 mm to −2.46 mm from Bregma, according to Paxinos and Franklin’s atlas.^24^ Brains were sliced using a Fisher Scientific NX-50 cryostat. Every sixth coronal section was selected, yielding ~ 8 sections per brain. The staining and mounting procedures were carried out as described.^10^ See **Table S1** for the antibodies used. TH- and Nissl- positive neurons in the SNpc were stereologically counted in a bright field via the optical fractionation method using a Zeiss AxioImager M2 microscope (Carl Zeiss, Cambridge, UK) and Stereo Investigator software (Version 10, MBF Bioscience, Magdeburg, Germany). Endpoints are presented as the total number of cells extrapolated based on the sample size. The size of the sampling grid covered 20% of the SNpc and area of each section, and the thickness is defined respectively by equations 1 and 2 (A= area, $t_{Q^{-}}^{-}$ average section thickness, $t_{i}=$ section thickness at site I, $Q_{i}=$ particles counted, m= number of sections).


1 $$A=\frac{1}{2}\left(x_{0}y_{n}-x_{n}y_{0}\right)+\sum_{i=1}^{n-1}\left(x_{i+1}y_{i}-x_{i}y_{i+1}\right)$$

2 $$t_{Q-}^{-}=\frac{\sum_{i=1}^{m}\;t_{i}Q_{i}^{-}}{\sum_{i=1}^{m}\;Q_{i}^{-}}$$

### 2.4 Western Blot (WB)

Following infusion of 20 μg of 6-OHDA into the dorsal striatum, mice were sacrificed at select time points after surgery, and brains were harvested, sub-dissecting out SNpc and striatum for both ipsilateral and contralateral sides. Tissue and samples were prepared as detailed elsewhere^23^ with the following exceptions: the boiling step was omitted, and Bolt 4% – 12% Bis-Tris-Plus gels were used for better separation of high-molecular-weight proteins (see **Table S1** for all markers and respective primary antibodies). The blots were imaged using the Sapphire Molecular Imager and analyzed with Azure Spot software (Azure Biosystems). Gel analysis was conducted using three control samples in each gel. Protein expression was normalized to a loading control—α-tubulin or glyceraldehyde 3-triphosphate dehydrogenase (GAPDH). Protein markers were analyzed for phosphorylation status using phosphorylation state-specific antibodies, and the phosphorylated protein level was reported as a ratio relative to the total protein level.

### 2.5 48-plex mouse cytokines/chemokines assay

Mice were deeply anesthetized, followed by intracardial puncture and perfusion with 1xPBS. Ventral midbrains were collected, and the SNpc was sub-dissected. The 48-plex mouse ProcartaPlex immunoassay (Thermo Fisher Scientific cat.n.: EPX480-20834-901) was performed following the manufacturer’s instructions and as published.^23^ Tissue was weighed and homogenized with a 1:10 ratio in lysis buffer containing 25 mM Tris-HCl, 100 mM NaCl, Halt protease inhibitor cocktail (1:100), phenylmethanesulfonyl fluoride (1:100), Triton X-100 (1:100), and NP-40 (1:100). The protein concentration was determined using a Pierce BCA assay kit (Thermo Fisher Scientific) and samples were diluted to obtain a protein concentration of 2 μg/μL. Samples were run in singlicate, and mean fluorescence intensity values obtained from 6-OHDA groups were transformed as log_2_ fold changes relative to the vehicle group for comparison.

### 2.6 Immunostaining and clearing (iDISCO+)

Immunolabelling and iDISCO + procedures followed published protocols^25^ with slight modifications. Antibodies used are listed in **Table S1**. Ten mice were infused with 6-OHDA (20 μg), and 8 mice were infused with the vehicle. All mice were infused in the right dorsal striatum. Two weeks later, mice were anesthetized (isoflurane 4%) and intracardially perfused with PBS, followed by 4% PFA in PBS. Brains were post-fixed overnight, washed 3 times in PBS with constant orbital shaking at room temperature (RT), and then dehydrated with methanol (45-minute incubations with 20, 40, 60, 80, and 100% methanol in PBS). After 1 hour in 100% methanol, brains were chilled to 4°C and transferred to a solution of dichloromethane: methanol (66:33) overnight at RT. Brains were washed 4 times in 100% methanol and bleached in methanol containing 5% hydrogen peroxide. Brains were rehydrated by reversing the methanol dilution series, washed for 1 hour with PBS and 0.2% Triton X-100, permeabilized (incubated in PBS containing 20% DMSO, 0.3 M glycine, and 0.16% Triton X-100 for 2 days), washed 2x with PTwH (PBS, 0.2% [v/v] Tween-20, 0.01% [w/v] heparin, and 0.02% sodium azide [w/v]), blocked (incubated in PBS containing 6% donkey serum and 10% DMSO for 4 days) and incubated in blocking buffer containing the primary antibody (**Table S1**) for 7 days. After repeated washes for three days, brains were incubated with the secondary antibody (**Table S1**). After three days of washing, brains were embedded in 1% [w/v] low melting point agarose (ThermoFisher; R0801; dissolved in 1xPBS, 0.001% [w/v] heparin, and 0.02% [w/v] sodium azide), dehydrated again, incubated overnight in dichloromethane: methanol (66:33) at RT, incubated twice in dichloromethane for 15 minutes, and cleared/stored in an airtight tube containing dibenzyl-ether.

### 2.7 Light sheet microscopy (LSFM)

After immunostaining and clearing, brains were imaged in 3D as described by Rijsketic et al.^21^ Briefly, agarose-embedded brains were attached to a c-clamp sample holder and lowered into an imaging chamber (Translucence Biosystems) filled with ethyl cinnamate. A Zeiss Lightsheet 7 controlled via Zen Black (3.1) was used to acquire tiled z-stacks spanning the whole brain (10% tile overlap), which were later stitched with Zen Blue (3.3). Pivot scanning and averaging of left and right light sheets (11 μm thick) minimized shadows and evened out illumination, respectively. 2D images were acquired using a 2.5x detection objective and 0.52x zoom, resulting in an x and y resolution of 3.5232 μm. Voxels were ~ isotropic with z-steps of 3.5 μm. Limiting the field of view to ~ 800×688 pixels optimized uniformity of illumination and resolution in the z dimension. 488 nm light (15% of 30 mW; 505–530 nm emission) excited autofluorescence for brain registration, while 561 nm light (3% of 75 mW; mirror: SBS LP 560; 585 nm LP emission) excited TH immunofluorescence (TH-IF). The exposure time for both wavelengths was 50 ms.

iDISCO+/LSFM data was analyzed using UNRAVEL (https://b-heifets.github.io/UNRAVEL/).^21^ Code refactored to Python can be accessed here (github.com/b-heifets/UNRAVEL/). In certain cases, older scripts were used (https://github.com/b-heifets/UNRAVEL/releases/tag/v0.1.2-feature-branch). Information on older code is available here (github.com/b-heifets/UNRAVEL/blob/feature/Heifets_lab_guides/UNRAVEL_guide_Heifets_lab_021623.pdf). Briefly, autofluorescence images were registered with an iDISCO/LSFM template (aligned with the Allen brain atlas), and immunofluorescence images were background subtracted and warped to atlas space for 2×2 voxel-wise ANOVAs. Significant voxels in f-statistic p-value maps (α = 0.05) were evaluated further with voxel-wise *post hoc* t-tests. The resulting p-value maps were false discovery rate (FDR) corrected using the most stringent q value yielding clusters of significant voxels. Clusters were warped to full-resolution tissue space for TH + cell or fiber density measurements. Clusters were considered valid if there was a difference in the density of TH + cells or fibers (two-tailed unpaired t-tests). Segmentation of TH + cells and fibers was performed with Ilastik. **Table S2** summarizes voxel-wise *post hoc* contrasts, effect directions, FDR q values, the adjusted p-value threshold, and cluster validation rates.

#### 2.7.1 Atlas brain registration (UNRAVEL)

Brain-wide quantification of fluorescent labels involves registration of the 3D autofluorescence image with an averaged template brain. We used a template from iDISCO+/LSFM aligned with the common coordinate framework version 3 (CCFv3) of the Allen brain atlas (ABA; atlas.brain-map.org/).^26^ We resampled this atlas and template to 25-micron resolution and lowered high-intensity IDs. Stitched image volumes were 3D cropped to minimize file sizes. Voxels outside the brain in the autofluorescence channel were zeroed out by setting the min and max of the display range and linearly scaling intensities within this range using Fiji.^27^ In the process, images were converted from .czi to a TIFF series. The autofluorescence image was downsampled by a factor of two, reoriented, and saved as a NIfTI image. If needed, additional voxels were masked with 3D Slicer (4.11 with the SegmentEditorExtraEffects extension). A modified version of MIRACL’s registration script^28^ was used to align the autofluorescence image with the average template and the corresponding atlas. We adjusted it to utilize the iDISCO+/LSFM-based template and atlas^26^, while also preventing image artifacts caused by bit depth changes and avoiding inaccurate brain masking. Registration accuracy was visually assessed by overlaying a 10 μm resolution version of the autofluorescence image with an aligned atlas image in ITK-SNAP (3.6.0). For three samples, better localization of the locus coeruleus during registration was manually fine-tuned using 3D Slicer.

#### 2.7.2 Voxel-wise statistics (UNRAVEL)

TH-IF image volumes were rolling ball background subtracted using a pixel radius of 20, increasing the sensitivity of voxel-wise analyses by only preserving signal from labeled cells and fiber tracts.^21^ Transformations from registration were applied to warp TH-IF images from tissue space to atlas space.^28^ After confirming correct warping using FSLeyes (part of the FMRIB Software Library [FSL]), TH-IF images were spatially smoothed to improve the overlap of voxel-wise signals across samples by convolving them with a Gaussian kernel (using fslmaths -s 0.05).^29^ The left hemispheres were flipped for comparisons with the right hemispheres. Voxels external to atlas labels or inside ventricles, the olfactory bulb, or undefined regions were excluded from voxel-wise comparisons.

To map voxels with potential differences in TH-IF, the randomise_parallel function from FSL carried out voxel-wise, GLM-based, non-parametric, permutation testing according to a 2×2 ANOVA design (18,000 permutations). Voxels reaching significance (uncorrected p < 0.05) for the main effect of side, the main effect of treatment, or interactions between these factors were further evaluated with *post hoc* voxel-wise t-tests (18,000 permutations). These pair-wise comparisons were made: ipsilateral vs. contralateral for 6-OHDA-treated mice, ipsilateral vs. contralateral for vehicle-treated mice, 6-OHDA vs. vehicle for the ipsilateral side, and 6-OHDA vs. vehicle for the contralateral side. To control for type-I errors, statistical stringency was adjusted using false discovery rate (FDR) correction^30^ as per Eq. (1). For each contrast, we selected q values that provided the maximum allowable stringency while permitting clusters of at least 100 significant voxels to survive, as described previously.^21^ Although increasing specificity in this way can reduce sensitivity—potentially leading to type-II errors—we chose to prioritize highlighting data in which we have the highest confidence. Statistical comparisons, effect directions, FDR q values, adjusted p-value thresholds, and cluster validation rates are listed in **Table S2**.

##### FDR procedure^30^:

1. Order voxel-wise p-values from smallest to largest.
2. Sequentially check if each p-value with meets the following constraint, starting with the least significant (largest) p-value:

$$p_{\left(i\right)}\leq \frac{i}{m}\times q\;\left(1\right)$$

where:

$p_{\left(i\right)}$ is the n^th^ p value,

i is the index of the n^th^ p-value,

m is the total number of voxel-wise comparisons,

q is the FDR q value

The adjusted p-value threshold is identified by the first p-value that meets this condition.

#### 2.7.3 Cluster validation (UNRAVEL)

Cluster validation was based on prior work.^21^ Clusters of voxels surviving FDR correction were partitioned for TH^+^ cell density measurements in regions with dopamine or norepinephrine neurons and TH^+^ fiber density measurements in the remainder of clusters. Clusters were warped to full-resolution tissue space for density measurements. Clusters were cropped and binarized for volume calculations. Cells and fibers were segmented in raw immunofluorescence images using Ilastik, where random forest classifiers were trained on all features by annotating 3 samples per condition (12 slices per label).^31^ Cell or fiber segmentations were multiplied by cluster masks to nullify external voxels. Cells were counted with CLIJ’s 3D objects counter on the GPU (2.2.0). TH^+^ fiber density was defined as ((# of voxels segmented as TH^+^ fibers) / (total # of voxels in the cluster))*100.^32^ Clusters were considered valid if an unpaired, two-tailed t-test demonstrated a significant difference in TH^+^ cell or fiber density between hemispheres or treatment groups. Sunburst plots were made using Flourish, and 3D models of valid clusters were created using DSI-Studio. For region-wise cell density measurements, the ABA was warped to tissue space (10 μm resolution), scaled to full resolution, and multiplied by the binary cell segmentation to enable 3D counting with respect to region labels.

### 2.8 Statistical analysis

Statistical analyses of lesion extent, western blots, and cytokines/chemokines were performed using GraphPad Prism software. ROUT (Q = 1%) was used to identify statistical outliers, which were subsequently excluded from analyses. Dunnett’s multiple comparison test was used to compare each 6-OHDA group or timepoint to the vehicle condition, while Šídák’s multiple comparison test was applied to compare 6-OHDA and vehicle groups for Luminex markers on each day evaluated. Values of *p* < 0.05 were considered statistically significant.

## 3 Results

### 3.1 The brain-wide distribution of 6-OHDA neurotoxicity in TH + cells and their projections

**Figure 2:**
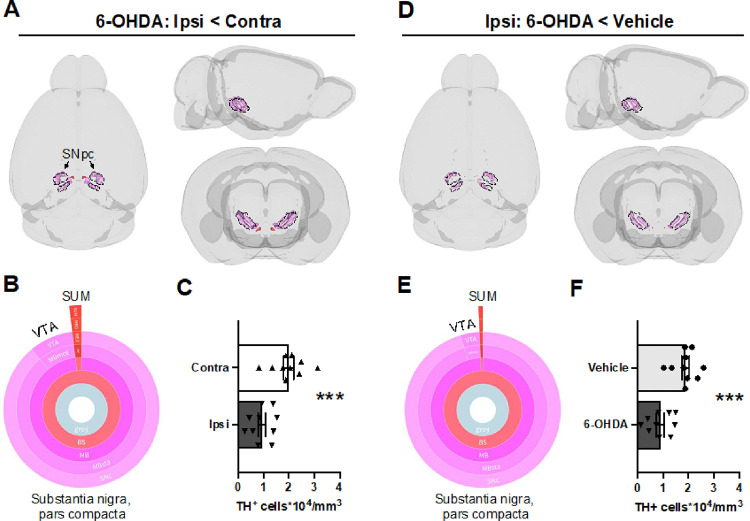
Mapping TH^+^ cell loss from 6-OHDA (20 μg; 2 weeks post infusion) in dopaminergic regions. Decreased TH-IF was mapped via voxel-wise comparisons of **A**) hemispheres ipsilateral (ipsi) and contralateral (contra) to the lesion or **D**) hemispheres ipsilateral to 6-OHDA or vehicle infusion. For display purposes, unilateral clusters were mirrored in 3D brain models. **A, D**) For both statistical contrasts, one cluster within regions containing dopamine neurons survived FDR correction (q < 0.0005), and it had a decreased density of TH+ cells. The predominant region with TH+ cell loss was the SNpc (89–95%), though a small portion of these clusters overlapped with the ventral tegmental area (VTA, 4–9%) and the supramammillary nucleus (SUM, 1–2%). Region coloring followed Allen brain atlas conventions. **B, E**) Sunburst plots summarize the relative volumes of composite regions. The outer ring shows the finest level of anatomical granularity, whereas inner rings represent parent regions. **C, F**) Quantification of TH+ cell densities (unpaired t-tests). Regional volumes and the location of clusters are summarized in **Table S3,** and cell densities for each mouse (including un-infused shams) are provided in **Table S4**. Mean ± SEM. ***p < 0.001.

**Figure 3:**
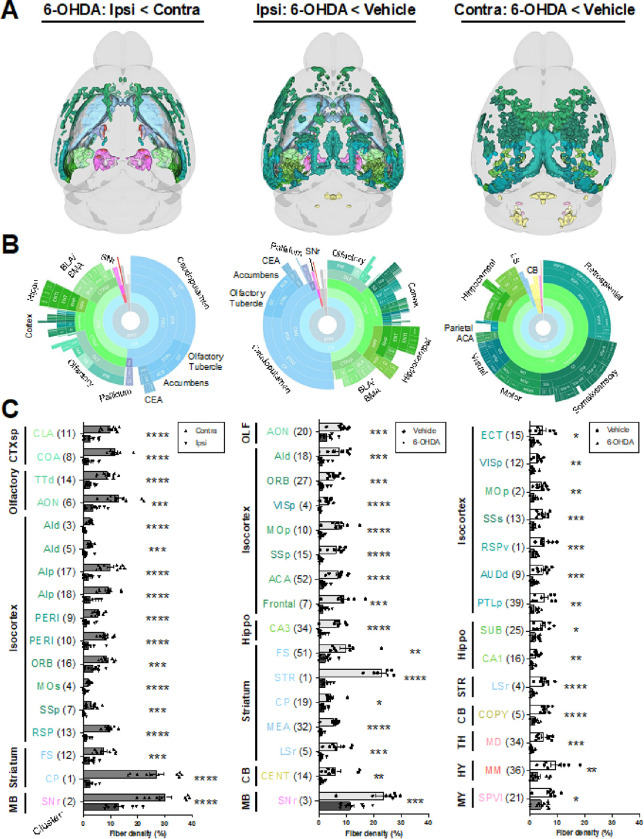
Decreased TH+ fiber densities from 6-OHDA (20 μg; 2 weeks post infusion). Clusters with decreased TH-IF from 6-OHDA after FDR correction and validation by fiber density measurements are shown (quantification for additional valid clusters is in **Figure S5**). Data are split into columns based on the comparison of post hoc voxel-wise t-tests. Left: decreased TH-IF in the ipsilateral (ipsi) hemisphere of 6-OHDA-treated mice compared to their contralateral (contra) hemisphere. Middle: decreased TH-IF in the ipsilateral hemisphere of 6-OHDA-treated mice compared to the ipsilateral hemisphere of vehicle-infused mice. Right: decreased TH-IF in the contralateral hemisphere of 6-OHDA-treated mice compared to the contralateral hemisphere of vehicle-infused mice. **A)** 3D brain models of valid clusters. **B)** Sunburst plots summarize regional volumes of valid clusters across levels of the Allen brain atlas hierarchy. **C)** Quantification of TH+ fiber density for each cluster. Fiber density was defined as: (volume of segmented voxels/total cluster volume)*100. Clusters were numbered in order of size but organized based on anatomy. The largest region of each cluster is depicted by its abbreviated name. Cluster locations, volumes, and regional compositions are summarized in **Table S3**, and raw fiber densities are in **Table S4.** Mean ± SEM. *p < 0.05, **p < 0.01, ***p < 0.001, ****p < 0.001. Abbreviations are defined in the ABA and the “Legend” tab of **Table S3.** Abbreviations that are not defined in **Table S3** include: d = dorsal; l = lateral; p = primary or posterior; r = rostral; s = secondary or supplemental; v = ventral.

Male mice were unilaterally infused in the dorsal striatum with 20 μg of 6-OHDA or the vehicle control, and 14 days later, their brains were fixed, immunostained for TH, optically cleared with iDISCO+, and imaged in 3D using LSFM. We utilized UNRAVEL to automate brain-wide mapping of TH immunolabeling and expanded its functionality to quantify changes in catecholaminergic cell groups and TH + fiber prevalence following 6-OHDA treatment. For this, autofluorescence images were registered with an iDISCO+/LSFM-derived average template brain aligned to the Allen brain atlas (ABA; CCFv3 2017), and TH-immunofluorescence (TH-IF) images underwent background subtraction and warping to atlas space for voxel-wise statistical analysis (**Fig. S1**). Out of the 9,040,331 voxels analyzed using a 2×2 ANOVA design, 5,787,716 voxels displayed significance for main effects and/or interactions, prompting further examination through voxel-wise *post hoc* t-tests. The resulting p-value maps were corrected for multiple comparisons using the FDR method to identify clusters of significant voxels. These clusters were partitioned based on their overlap with dopaminergic and noradrenergic cell groups. TH + cell density measurements were taken within these regions, and fiber densities were assessed outside these regions (**Figs. S1** and **S2**). For validation, clusters were transformed to full-resolution tissue space for precise cell or fiber density measurements using segmentations from Ilastik. Clusters with significant differences between conditions (either side or treatment group) for these measurements were considered valid. As anticipated, TH-IF was reduced in the SNpc and regions it innervates (Fig. 2–3 and **Fig. S2**, **S3**, and **S5**). **Table S3** provides information on regional abbreviations and valid clusters’ significance, volumes, position, and regional composition. Raw TH + cell and fiber densities and p-values are provided in **Table S4**. Fly-through videos created with Imaris showcase representative examples of TH-IF in lesioned (**Video S1**) and control (**Video S2**) mice.

In regions housing dopamine neurons, only one cluster reflected TH + cell loss when comparing the hemispheres of 6-OHDA-treated mice or the ipsilateral hemisphere of treatment groups (Fig. 2; q < 0.0005). In both cases, the loss of dopamine neurons was primarily concentrated in the SNpc (6-OHDA: ipsilateral vs. contralateral, p = 0.00049; Ipsilateral: vehicle vs. 6-OHDA, p = 0.00062). Consistent with these findings, region-wise TH + cell density measurements in dopaminergic cell groups also indicated selective loss of neurons in the SNpc (p < 0.0015; Tukey’s post-test comparing contralateral and ipsilateral sides of 6-OHDA-infused mice; **Fig. S3**). No TH + cell loss was observed in noradrenergic regions with either cluster-wise or region-wise analyses (**Fig. S4**).

**Figure 4:**
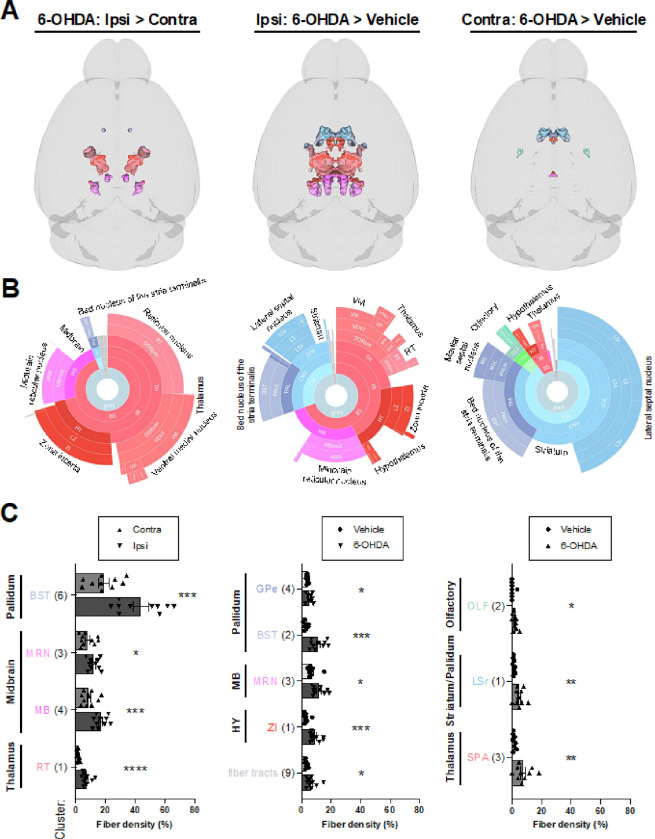
Increased TH+ fiber densities from 6-OHDA (20 μg; 2 weeks post-infusion). Clusters with increased TH-IF from 6-OHDA after FDR correction and validation by fiber density measurements are shown. Data are split into columns based on the comparison of *post hoc* voxel-wise t-tests. Left: increased TH-IF in the ipsilateral (ipsi) hemisphere of 6-OHDA-treated mice compared to their contralateral (contra) hemisphere. Middle: increased TH-IF in the ipsilateral hemisphere of 6-OHDA-treated mice compared to the ipsilateral hemisphere of vehicle-infused mice. Right: increased TH-IF in the contralateral hemisphere of 6-OHDA-treated mice compared to the contralateral hemisphere of vehicle-infused mice. **A**) 3D brain models of valid clusters. **B**) Sunburst plots summarize regional volumes of valid clusters across levels of the ABA hierarchy. **C**) Quantification of TH+ fiber densities for each cluster. The largest region of each cluster is noted. Cluster locations, volumes, and regional compositions are summarized in **Table S3,** and raw fiber densities are in **Table S4**. Mean ± SEM. *p < 0.05, **p < 0.01, ***p < 0.001, ****p < 0.001. Abbreviations are defined in the ABA and the “Legend” tab of **Table S3**. Other abbreviations include: r = rostral.

6-OHDA induced widespread loss of TH + fibers in both the infused and contralateral hemispheres (Fig. 3 and **Fig. S5**). Ipsilateral fiber loss was most prominent in the striatum (e.g., caudoputamen, olfactory tubercle, and nucleus accumbens), olfactory regions (e.g., piriform cortex), isocortex, hippocampal formation, basolateral/basomedial amygdala, pallidum, and substantia nigra, pars reticulata (SNr). Fiber loss was predominantly cortical in the contralateral hemisphere, with notable decreases in retrosplenial, somatosensory, motor, visual, and hippocampal areas. Compensatory increases in TH + fiber density were most evident on the ipsilateral side. However, they were also observed in a subset of the same regions in the contralateral hemisphere (Fig. 4). The primary regions displaying ipsilateral compensation included the thalamus (e.g., the reticular nucleus and ventral medial nucleus), zona incerta, and midbrain reticular nucleus. When comparing 6-OHDA and vehicle-treated brains, fiber density increased bilaterally in the lateral/medial septal nucleus, bed nucleus of the stria terminalis, hypothalamus, and thalamus. 3D patterns of TH + cell loss, TH + fiber loss, and upregulation of TH in projection sites are summarized in **Video S3**.

### 3.2 6-OHDA nigrostriatal lesion characterization – sex, dosage, and time course differences

To better understand the neurotoxic effects of 6-OHDA, we examined how dosage and sex influence the extent and progression of 6-OHDA-induced lesions. Lesion extent was assessed using western blotting to measure striatal TH protein levels, stereological counts of TH + cells, and mean fluorescent intensity of TH levels in the SNpc.

**Figure 5:**
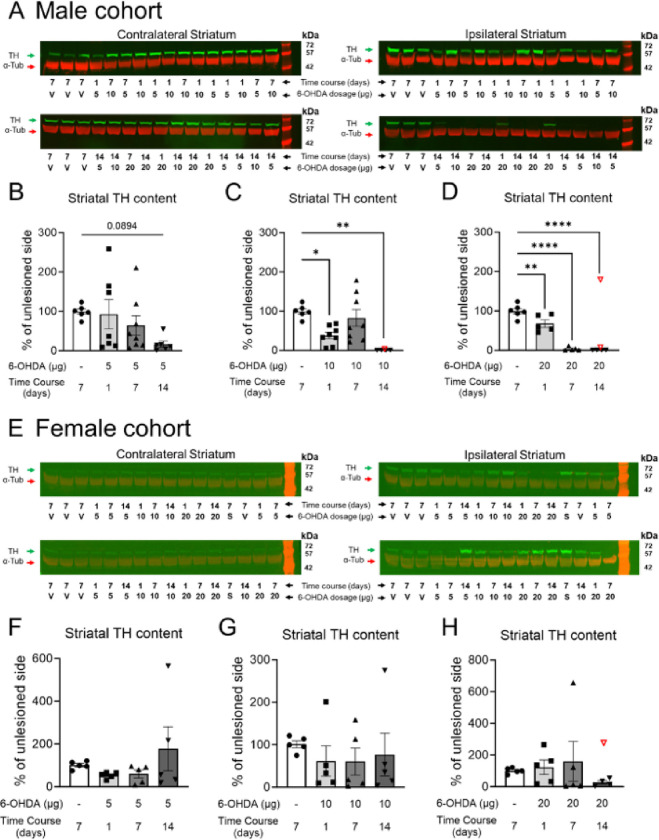
Striatal tyrosine hydroxylase (TH) levels following infusion of 5, 10, or 20 μg of 6-OHDA are reduced in male C57BL/6 mice. **A** and **E)** Representative western blot membranes probed for TH (green, molecular weight ≈ 58 kDa) and α-tubulin (red, molecular weight ≈ 50 kDa). TH protein levels serve as a proxy for striatal dopaminergic lesions. **B-D)** Quantification of TH protein levels in male cohorts for 5 μg, 10 μg, and 20 μg dosages of 6-OHDA, respectively. **F-H)** Quantification of TH protein levels in female cohorts for 5 μg, 10 μg, and 20 μg dosages of 6-OHDA, respectively. *p < 0.05, **p < 0.01, and ****p < 0.0001. The same vehicle group was used as a control across all 6-OHDA dosages. V = vehicle, S = sham. 19.5 μg of protein was loaded per well. Data are reported as mean ±SEM. **Group sizes:** Male cohort: Vehicle group: n = 6; 6-OHDA groups: 5 μg day 1 n = 7, day 7 n = 8, and day 14 n = 6; 10 μg day 1 n = 8, day 7 n = 8, and day 14 n = 5; 20 μg day 1 n = 5, day 7 n = 5, and day 14 n = 6. Female cohort: n = 5 for each group. Refer to **Fig. S6** and **Fig. S7** for full gel images and data normalized to the loading control α-tubulin. Excluded outliers (ROUT test) are shown as clear symbols with a red outline.

TH protein levels in the striatum of male mice showed a gradual decrease in dopaminergic fibers over 14 days following a 5 μg 6-OHDA infusion (Fig. 5A–B; **Fig. S6 P**), nearing significance only on day 14 compared to controls (p = 0.089). In contrast, the higher doses of 10 and 20 μg of 6-OHDA induced a significant depletion of TH fibers (10 μg: one-way ANOVA F_(3,22)_ = 7.513, p = 0.0012, 20 μg: F_(3,16)_ = 59.90, p < 0.0001) as early as 24 hours post-infusion (Fig. 5C–D; **Fig S6 Q-R**). Interestingly, female mice exhibited a reduced susceptibility to 6-OHDA toxicity in the striatum, particularly in the 5 μg and 10 μg groups, where striatal TH protein levels tended to decrease on day 1 and day 7, but no impact was detected on day 14 (Fig. 5E–H).

**Figure 6:**
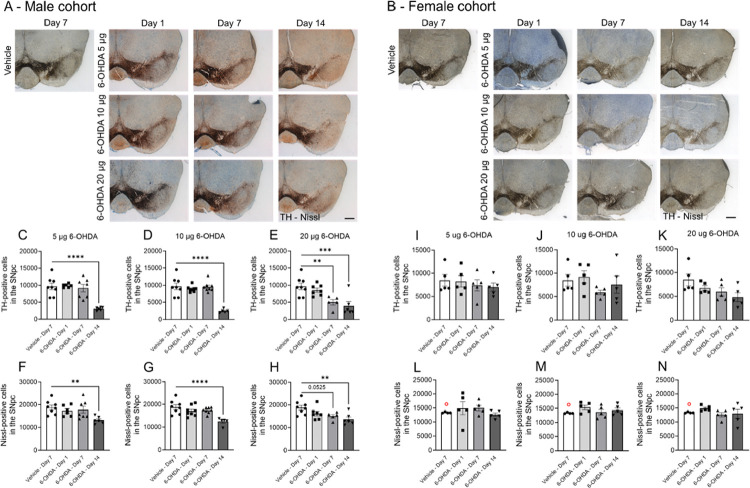
Optical fractionation cell counting of TH-positive cells and Nissl-positive neurons in the SNpc of male and female C57BL/6 mice infused with 6-OHDA. A series of example ipsilateral coronal sections immunohistochemically stained for TH (dark brown from DAB) and counterstained for Nissl (blue) show labeling of the VTA and SNpc for **A**) males and **B**) females. Sections were taken between −3.1 and −3.4 mm from Bregma. Scale bar = 500 μm. Male cohort: TH-positive cells for **C)** 5 μg, **D)** 10 μg, and **E)** 20 μg dosages of 6-OHDA, and Nissl-positive neurons for **F)** 5 μg, **G)** 10 μg, and **H)** 20 μg dosages. Female cohort: TH-positive cells for **I)** 5 μg, **J)** 10 μg, and **K)** 20 μg dosages of 6-OHDA and Nissl-positive neurons for **L)** 5 μg **M)** 10 μg and **N)** 20 μg dosages. Values are reported as the total number of cells in the ipsilateral SNpc. One-way ANOVAs were performed for each dose with Bonferroni *post hoc* comparisons between vehicle and 6-OHDA-infused groups. **p < 0.01, ***p < 0.001, and ****p < 0.0001. Only the ipsilateral SNpc was quantified due to the labor-intense and time-consuming nature of the experiment. Brightfield images were taken with a 2.5x objective (Zeiss EC Plan-Neofluar 2.5/0.075) mounted on an epifluorescence AxioImager M2 microscope. Data are reported as mean ± SEM. The same vehicle group was used as the control across all 6-OHDA dosages. Vehicle mice were culled on day 7 post-surgery. V = vehicle. **Group sizes:** Male cohort: vehicle n = 8; 6-OHDA groups: 5 μg day 1 n = 6, day 7 n = 7, and day 14 n = 6; 10 μg day 1 n = 8, day 7 n = 8, and day 14 n = 5; 20 μg day 1 n = 7, day 7 n = 6, and day 14 n = 7. Female cohort: n = 5 for each group. Excluded outliers (ROUT test) are shown as clear symbols with a red outline.

**Figure 7:**
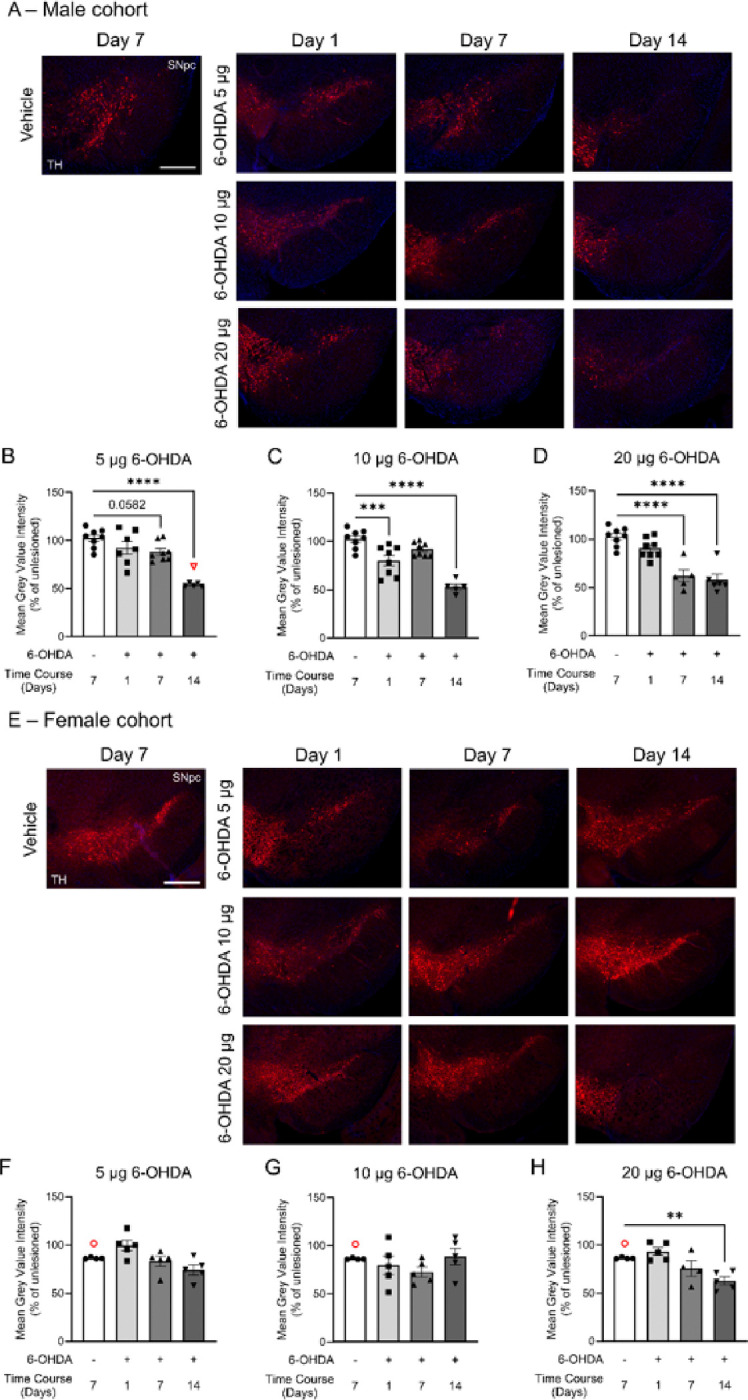
Time-course of TH-immunofluorescence labeling in the SNpc of male and female C57BL/6 mice exposed to three 6-OHDA dosages. Representative images of ventral midbrain slices from **A)** male and female mice show TH labeling (red) of somata in the SNpc and VTA, along with DAPI counterstaining (blue). Male cohort: TH mean fluorescence for **B)** 5 μg, **C)** 10 μg, and **D)** 20 μg dosages of 6-OHDA. Female cohort: TH mean fluorescence for **F)**5 μg, **G)** 10 μg, and **H)** 20 μg dosages of 6-OHDA. Mean fluorescence values were averaged using 2 sections per mouse brain on each side. Data are reported as percentage averages of the mean fluorescence intensity of the ipsilateral SNpc relative to the contralateral SNpc ((ipsi/contra)*100). Female controls had notably higher contralateral values compared to the ipsilateral side, suggesting that saline infusion may have modestly reduced TH levels. Datasets were analyzed using one-way ANOVAs with Bonferroni *post hoc* tests for pair-wise comparisons to the vehicle group. **p < 0.01, ***p < 0.001, and ****p < 0.0001. The same vehicle group was used as a control across all three 6-OHDA regimens. Ipsilateral and contralateral coronal ventral midbrain sections were taken approximately at −3.1 mm from Bregma. Images were taken with a 5x objective (Zeiss Fluar 5x/0.25) mounted on an epifluorescence AxioImager M2 microscope. Data are reported as mean ±SEM. Scale bar = 400 μm. **Group sizes:** Male cohort: vehicle n = 8; 6-OHDA groups: 5 μg day 1 n = 7, day 7 n = 8, and day 14 n = 6; 10 μg day 1 n = 8, day 7 n = 8, and day 14 n = 5; 20 μg day 1 n = 8, day 7 n = 5, and day 14 n = 6. Female cohort: n = 5 for each group. Excluded outliers (ROUT test) are shown as clear symbols with a red outline.

Concurrently, stereological counting of TH + neurons and quantifying mean fluorescent intensity in the SNpc were performed for male and female mice. Stereological counting revealed an abrupt, delayed loss of TH + cells in male mice following infusion of 5 μg or 10 μg of 6-OHDA at day 14 (Fig. 6A C–D, 5 μg: F_(3,23)_ = 14.55, p < 0.0001, 10 μg: F_(3,25)_ = 22.22, p < 0.0001). The 20 μg dose of 6-OHDA led to a more rapid decline in TH cell prevalence over time (F_(3,22)_ = 8.726, p = 0.0005), with significant reductions on day 7 and day 14 (Fig. 6A and E). The TH-, Nissl + neuronal population in the SNpc was similarly affected across the 6-OHDA dosages (one-way ANOVAs: 5 μg, p = 0.0124; 10 μg, p = 0.0006; 20 μg, p = 0.0024) (Fig. 6F–H). Measurement of mean fluorescence intensity in the SNpc in male mice showed a similar temporal pattern of TH loss across the 6-OHDA dosages when expressed as a percentage of the contralateral side (Fig. 7A–D). TH fluorescence was reduced by 5 μg and 10 μg dosages on day 14 (Fig. 7A–C, one-way ANOVAs: 5 μg, F_(3,24)_ = 18.80, p < 0.0001; 10 μg F_(3,25)_ = 23.97, p < 0.0001). The 20-μg dose of 6-OHDA accelerated the decrease in TH-IF, with reductions observed on days 7 and 14 (Fig. 7D). Thus, in male mice, 6-OHDA consistently reduced TH + cell abundance and TH expression in the SNpc at day 14 across all dosages, representing a decrease of ~ 75% for stereologically counted TH + cells (Fig. 6C–E) and ~ 50% for mean fluorescent intensity (Fig. 7B–D).

Stereological cell counting in the SNpc of female mice demonstrated their resilience to 6-OHDA toxicity, with none of the 6-OHDA dosages significantly affecting the TH + cell population (Fig. 6B, I–K). Similarly, the Nissl + cell population remained unaffected (Fig. 6L–N), and changes in the mean TH-IF intensity in the SNpc were modest (Fig. 7E–H). An overall drug effect was detected for the 5 μg (F_(3,15)_ = 5.073, p = 0.013) and 20 μg (F_(3,14)_ = 8.399, p = 0.0019) 6-OHDA dosages (Fig. 7E–H). While TH-IF labeling was reduced at day 14 (Fig. 7H), the reduction was not as pronounced as in male mice (Fig. 7D).

Both measurements in the SNpc generally showed similar temporal patterns of neurotoxicity across doses. They were significantly correlated with each other, with a Pearson’s correlation coefficient of 0.558 for males and 0.267 for females (**Table S5**). In male mice, there were small but significant correlations between the SNpc measurements and striatal TH protein levels. However, in female mice, striatal TH protein expression did not correlate with either histological endpoint, consistent with milder pathology.

### 3.3 6-OHDA triggers neuroinflammation before the onset of neuronal loss

**Figure 8:**
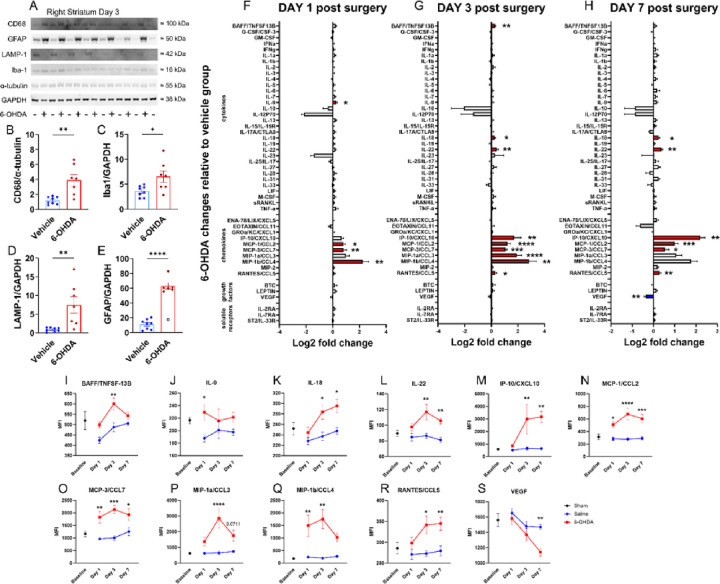
Striatal infusion of 20 μg of 6-OHDA altered inflammatory markers at the infusion site and in the SNpc. **A**) Three days after 6-OHDA infusion, inflammatory marker proteins in striatal lysates were analyzed using Western blotting. Each well was loaded with 19.5 μg of total protein. Densitometric quantification of **B)** CD68, **C)** Iba1, **D)** LAMP-1, and **E)** GFAP for vehicle and 6-OHDA groups, with expression normalized to GAPDH or α-tubulin (two-tailed unpaired t-tests). **F-H**) A Luminex immune monitoring panel quantified protein levels of 48 cytokines and chemokines in lysates from the SNpc at days 1, 3, and 7 post-infusion. The expression of immune proteins is represented by log_2_-fold changes in the mean fluorescence intensity (MFI) relative to vehicle groups (two-tailed unpaired t-tests). Red and blue bars represent significant upregulation and downregulation, respectively. **I-S**) For chemokines/cytokines with a change in expression, we plotted their baseline level and their expression in 6-OHDA- and vehicle-treated groups at each time point (pair-wise Bonferroni-corrected comparisons). *p < 0.05, **p < 0.01, ***p < 0.001, and ****p < 0.0001. Data are reported as mean ± SEM. Western blotting cohort: vehicle n = 8, 6-OHDA n = 7. An excluded outlier (ROUT test) is shown as a clear symbol with a black outline. Multiplex cytokine cohorts: day 1 vehicle n = 6, 6-OHDA n = 8; day 3 vehicle n = 6, 6-OHDA n = 6; day 7 vehicle n = 6, 6-OHDA n = 8.

In the region where 6-OHDA was infused, western blotting revealed clear evidence of inflammation three days later, as indicated by an upregulation of microglial markers CD68 (t = 3.77, df = 13, p = 0.0024), Iba1 (t = 2.60, df = 13, p = 0.022), and Lamp-1 (t = 3.23, df = 13, p = 0.0066) as well as the astrocytic marker GFAP (t = 11.72, df = 12, p < 0.0001) (Fig. 8A–E; **Fig. S8**). We analyzed 48 cytokines and chemokines in the SNpc with a 48-plex assay following infusion of 20 μg of 6-OHDA into the dorsal striatum of male C57BL/6 mice. The expression of 11 neuroinflammatory markers was altered, as indicated by Log2-fold change analysis, with tissue collection on days 1, 3, and 7 following 6-OHDA infusion (Fig. 8F–H). Multiple chemokines were upregulated across time points, indicating the consistency of these measurements. The effect of 6-OHDA on cytokines was analyzed using a mixed-effect model, followed by *post hoc* Šídák’s correction for multiple comparisons. Figure 8I–S details the chemokines/cytokines that were significantly altered for at least one time point. A sustained increase in expression was observed for MCP-1/CCL2, MCP-3/CCL7, and MIP-1β across time points. Other markers were increased at the latter two-time points (IL-18, IL-22, IP-10/CXCL10, and RANTES/CCL5). IL-9 was only increased on day 1, and BAFF/TNFSF-13B and MIP-1a/CCL3 were only increased on day 3. VEGF was the only cytokine that decreased significantly (day 7). The upregulation of MCP-1/CCL2, MCP-3/CCL7, and MIP-1β may drive immune cell recruitment, microglial proliferation, and inflammatory gene expression. Delayed increases in IL-18, IL-22, IP-10/CXCL10, and RANTES/CCL5 suggest a progression of chronic neuroinflammation, while early IL-9 and mid-stage BAFF/TNFSF-13B and MIP-1a/CCL3 increases may reflect an evolving response. The decrease in VEGF at day 7 may improve blood-brain barrier integrity and aid the resolution of immune cell recruitment.

After profiling the inflammatory response in the nigrostriatal tract, we investigated the phosphorylation status of multiple kinases in the striatum seven days after a 20 μg 6-OHDA infusion to examine the engagement of specific biochemical signaling pathways that regulate apoptosis. Death-associated protein kinase 1 (DAPK1), which is typically auto-phosphorylated at Ser308 to autoinhibit its CaM binding site and remain inactivated, showed a reduction in the ratio of phosphorylated DAPK1 (pDAPK1) to total DAPK (t = 2.34, df = 15, p = 0.033; **Figs. S9 A** and **B; S10**). This reduction indicates local excitotoxicity and/or pro-apoptotic signaling in the axons of SNpc neurons.^33,34^ Conversely, phosphorylation of GSK-3β at Thr390 relative to total GSK-3β was increased (**Figs. S9 A** and **C; S10**), suggesting that it was inactive and did not promote pro-apoptotic signaling. Phosphorylation of AKT at Ser473 increased slightly but not significantly, which might reflect a modest role in promoting cellular survival. Together, these findings characterize the profile of inflammatory and apoptotic signaling induced by 6-OHDA toxicity.

## 4 Discussion

In this study, we investigated the histopathological effects of 6-OHDA infusion into the striatum of C57BL/6 mice. Using a novel image analysis technique to map brain-wide changes in TH expression and relate these to TH + neuron and projection densities, we found that neurodegeneration from 20 μg 6-OHDA at 14 days post-infusion was confined to the SNpc and associated with widespread, bilateral denervation of its striatal and extra-nigrostriatal targets, along with compensatory TH upregulation in subcortical regions. Male mice showed more severe pathology, indicating potential neuroprotective mechanisms in females. In male mice, lower doses (5 and 10 μg) led to a delayed reduction in striatal TH expression, primarily at day 14, while the highest dose (20 μg) caused a progressive, severe decrease in dopaminergic fibers in the striatum. Both optical fractionation and mean fluorescence intensity reliably indicated TH + cell loss in the SNpc, validating the latter as an efficient alternative to stereological counting. We observed robust glial activation (CD68, Iba1, LAMP1, GFAP) in the striatum three days after 6-OHDA infusion, along with an upregulation of inflammatory markers in the SNpc that began within a day and primarily sustained for at least a week preceding morphological cell death. Additionally, we found that 20 μg of 6-OHDA activated DAPK1 in the striatum, potentially promoting excitotoxicity, while GSK-3β and AKT did not regulate pro-apoptotic signaling.

We demonstrated that the 6-OHDA lesion can be extensively characterized with light-sheet fluorescence microscopy (LSFM) and quantitative 3D analysis of TH + neurons. Likewise, Roostalu, et al. observed a similar, albeit milder, pattern of TH loss after systemic injection of MPTP.^35^ Our catecholaminergic mapping data aligns with the conventional WB and 2D quantification techniques used in this and other studies.^36,37^ For example, 6-OHDA decreased the density of TH + cells 51–52% in a singular cluster localized 89–95% to the SNpc, 4–9% to the VTA, and 1–2% to the supramammillary nucleus (SUM). Conversely, loss of TH + fibers was widespread, impacting the striatum, isocortex, midbrain, hippocampal formation, olfactory areas, and amygdalar nuclei. As TH + fiber loss spreads beyond regions with dopaminergic innervation—such as the striatum, amygdala, hippocampus, prefrontal cortex, anterior cingulate area^38^, and rhinal cortices^39^—damage also affects noradrenergic projections. These projections extend widely across the brain, reaching areas including the isocortex, thalamus, hypothalamus, hippocampus, amygdala, and cerebellum.

In contrast to the widespread TH + fiber loss, compensatory increases were spatially confined to a few subcortical regions, including the thalamus, hypothalamus, midbrain, pallidum, and lateral septal nucleus. Consistent with this, bilateral remodeling of efferents and afferents connected to the dopaminergic A13 cell group in the hypothalamus has been reported following a unilateral infusion of 6-OHDA (3.6 μg) into the medial forebrain bundle, with mapping occurring 29 days later.^40^ Although the paraventricular hypothalamus (PVH), dopaminergic A13 group, the nucleus of reuniens (RE), and pontine central gray (PCG) appeared to show compensatory increases in TH + cell density, this likely indicates enhanced TH expression and/or TH fiber density, leading to the heightened detection of TH + cells.^11,41,42^ Interestingly, nuclei with noradrenergic neurons, such as the locus coeruleus (LC), were resistant to 6-OHDA toxicity. These regions may activate a compensatory response to counteract the catecholaminergic imbalance caused by 6-OHDA in the striatum.^43^ Given the projection patterns of noradrenergic neurons, the increased TH levels in regions with compensation may indicate heightened noradrenergic tone, mitigating the effects of reduced dopamine levels.^44^

Our brain-wide mapping enhances our understanding of regions implicated in motor and non-motor impairments in PD, extending beyond the traditional focus on nigrostriatal denervation. By leveraging machine learning and employing analytical tools from fMRI used in PD patients,^45^ our 3D analysis of immunolabeling in rodent brains efficiently screens anatomically analogous regions, identifying areas relevant to different PD stages and symptoms. Moreover, our approach provides a framework for spatially resolving neuronal morphology, glial reactivity, and disease markers.

Only two observational studies have explored the use of 6-OHDA in female C57BL/6 mice,^11,18^, with Masini and colleagues being the only ones considering sex differences at the histopathological level.^11^ Consistent with the findings of Masini et al., our results demonstrated a remarkable resistance of female mice to all doses of 6-OHDA. This resistance may be partially attributed to estrogen, which is known to support elevated levels of brain-derived nerve factor.^46^ It might also contribute to the lower incidence of PD in women. However, further investigation is needed to explore the neuroprotective mechanisms in female mice that underlie their resilience against the 6-OHDA neurotoxin at the doses studied here. Based on data from this and other studies, we strongly recommend conducting *a priori* power calculations when designing PD research involving female mice.^11,18^

In our study, the highest 6-OHDA dosage progressively reduced TH protein levels in the striatum and the prevalence of TH + cells in the SNpc within 14 days. Other studies have delivered 18 or 20 μg of 6-OHDA in the striatum, with results showing a 40% degeneration of the SNpc in one case^17^ and a more severe 60% reduction in another at 14 days.^47^ Considering the timing of TH downregulation and SNpc neuron loss, selecting the appropriate 6-OHDA dose is crucial based on the research question and histopathological endpoints. The lowest dose (5 μg) causes a gradual reduction in striatal TH + expression, which may not reach significance but still leads to SNpc neuron loss by day 14, indicating a milder injury, which could be easier to rescue. This makes it a sensitive readout for testing neurotherapeutics aimed at stabilizing pre-synaptic terminals or exploring striatal mechanisms, provided the studies are sufficiently powered. In contrast, the highest dose (20 μg) rapidly reduces TH + neurons in the SNpc within 7 days, making it better suited for efficient screening of experimental therapies attempting to preserve cell bodies and slow rostrocaudal degeneration of dopaminergic neurons. These findings should guide the dose selection in studies targeting specific neuroprotective strategies.

The optical fractionator stereology cell counting method is considered the gold standard for quantification of dopaminergic lesions in the SNpc.^48–50^ However, mean fluorescent intensity measurements have also been successfully employed in several studies,^18,51^ producing comparable results. Our data reinforces using mean fluorescent intensity as a proxy for TH cell densities, a more time-efficient alternative to the labor-intensive stereology cell counting method.

While many studies have documented astrocyte and microglial reactivity in rats and mice treated with 6-OHDA,^17,52,53^ none have previously examined cytokine/chemokine levels using a 48-plex immunoassay. Here, we report a distinct chemokine/cytokine signature in the SNpc on days 1, 3, and 7 following 6-OHDA infusion. The robust upregulation of chemokines from 6-OHDA is likely driven by proinflammatory, activated microglia, which promote chemoattraction (e.g., via CCL5) of CD8 + T helper cells, eosinophils, and monocytes. In turn, chemokines such as MIP-1α, MIP-1β, and MIP-2 create a sustained positive feedback loop of proinflammatory microglial activation and proliferation.^54,55^ IP-10/CXCL10 signaling leads to the activation of apoptotic pathways,^56^ while upregulation of the cytokine BAFF/TNFSF13B, primarily released by B cells, aligns with evidence of B and T cell infiltration in the brains of 6-OHDA-infused mice.^57^ This also explains the increase in RANTES/CCL5, predominantly related to T-cell infiltration.^57^ Our observation of a robust, rapid inflammatory response suggests early activation of microglia and astrocytes driven by damaged SNpc neurons.^17^ Glial reactivity is further evidenced by the increased protein levels of CD68, Iba1, LAMP1, and GFAP in the striatum on day 3, although the mechanisms underlying activation at the infusion site may differ. Notably, the cytokine fingerprint detected in the 6-OHDA model is not entirely detrimental; for example, RANTES/CCL5 has shown neuroprotective effects in Alzheimer’s disease,^58^ while RANTES and MIP-1β have potent recruitment activity toward T lymphocytes, which are protective in 6-OHDA-treated mice.^59^ In contrast, the cytokine profile in PD patients is substantially different, with increased levels of TNF-α, IL-1β, IL-2, IL-4, IL-6, TGF-α, TGF-β1, and TGF-β2.^60^ These differences suggest that the 6-OHDA mouse model may be ideal for developing and testing immunomodulatory therapies aimed at rescuing SNpc neurons through the modulation of chemokine and cytokine expression.

We also examined the expression and phosphorylation levels of kinases (DAPK1, GSK-3β, and AKT) that regulate apoptosis.^61,62^ DAPK1 activity was increased in the striatum of 6-OHDA-treated mice at day 7, potentially indicating ongoing apoptosis due to excitotoxicity, which is associated with neuronal cell death in both *in vivo* and *in vitro* models.^33^ In contrast, GSK-3β and AKT were not activated, preventing them from influencing neuronal degeneration and survival.

In summary, this study provides a detailed characterization of the 6-OHDA mouse model, incorporating comprehensive catecholaminergic mapping, three different time points and dosages of 6-OHDA, and the inclusion of both male and female mice. Traditional histopathological methods effectively detected dose-dependent, progressive striatal TH + fiber loss and SNpc neurodegeneration, while also revealing resilience in female mice. These findings were complemented by an innovative approach for quantifying brain-wide iDISCO+/LSFM data. Specifically, differential TH immunolabeling was spatially resolved through voxel-wise analyses and carefully evaluated with FDR correction and precise measurements of TH + cell and fiber densities in the most affected regions. This study also highlights the early immune response preceding neuronal cell death in the striatum and SNpc, revealing a distinct cytokine/chemokine signature to that observed in PD patients. Additionally, we identified increased DAPK1 activity, which may drive excitotoxicity in SNpc neurons. Our findings offer valuable insights into the 6-OHDA mouse model, delineating brain-wide histopathology and the biochemical signaling profile in the nigrostriatal pathway. Furthermore, our methodologies can be applied to other animal models of neurodegenerative diseases, potentially advancing our understanding of disease processes and the development of new therapeutic strategies.

## Data Availability

The data and scripts that support the findings of this study are downloadable from the following link (OSF repository): https://osf.io/kmp29/?view_only=368db4f5e49c4737a6a7b13528a559e4 Code used for this study is also available at https://zenodo.org/records/13377341 and https://github.com/b-heifets/UNRAVEL/releases (tag: v0.1.2-feature-branch).
